# Artificial intelligence could alert for focal skeleton/bone marrow uptake in Hodgkin’s lymphoma patients staged with FDG-PET/CT

**DOI:** 10.1038/s41598-021-89656-9

**Published:** 2021-05-17

**Authors:** May Sadik, Jesús López-Urdaneta, Johannes Ulén, Olof Enqvist, Armin Krupic, Rajender Kumar, Per-Ola Andersson, Elin Trägårdh

**Affiliations:** 1 Department of Molecular and Clinical Medicine, Clinical Physiology, Sahlgrenska University Hospital, Sahlgrenska Academy at the University of Gothenburg, 413 45, Gothenburg, Sweden; 2 Eigenvision AB, Malmö, Sweden; 3 Department of Electrical Engineering, Chalmers University of Technology, Gothenburg, Sweden; 4 Department of Nuclear Medicine, Post Graduate Institute of Medical Education and Research, Chandigarh, India; 5 Department of Haematology, Södra Älvsborg Hospital, Borås, Sweden; 6 Clinical Physiology and Nuclear Medicine, Skåne University Hospital, Malmö, Sweden

**Keywords:** Haematological diseases, Molecular medicine

## Abstract

To develop an artificial intelligence (AI)-based method for the detection of focal skeleton/bone marrow uptake (BMU) in patients with Hodgkin’s lymphoma (HL) undergoing staging with FDG-PET/CT. The results of the AI in a separate test group were compared to the interpretations of independent physicians. The skeleton and bone marrow were segmented using a convolutional neural network. The training of AI was based on 153 un-treated patients. Bone uptake significantly higher than the mean BMU was marked as abnormal, and an index, based on the total squared abnormal uptake, was computed to identify the focal uptake. Patients with an index above a predefined threshold were interpreted as having focal uptake. As the test group, 48 un-treated patients who had undergone a staging FDG-PET/CT between 2017–2018 with biopsy-proven HL were retrospectively included. Ten physicians classified the 48 cases regarding focal skeleton/BMU. The majority of the physicians agreed with the AI in 39/48 cases (81%) regarding focal skeleton/bone marrow involvement. Inter-observer agreement between the physicians was moderate, Kappa 0.51 (range 0.25–0.80). An AI-based method can be developed to highlight suspicious focal skeleton/BMU in HL patients staged with FDG-PET/CT. Inter-observer agreement regarding focal BMU is moderate among nuclear medicine physicians.

## Introduction

Skeleton/bone marrow involvement in patients with newly diagnosed Hodgkin’s lymphoma (HL) is an important predictor of adverse outcomes^1^. Studies show that FDG-PET/CT upstages patients with uni- or multifocal skeleton/bone marrow uptake (BMU) when iliac crest bone marrow biopsy fails to find evidence of histology-proven involvement^2,3^. The general recommendation is, therefore, that bone marrow biopsy can be avoided when FDG-PET/CT is performed at staging^4,5^.

Classifications of focal skeleton/BMU can sometimes be challenging. Artificial intelligence (AI) is being developed to assist the observer by highlighting suspicious uptake in diagnostic images^6^. Earlier studies have showed that AI can be trained to find suspicious bone involvement and provide the results quantitatively and objectively, rather than in a subjective way^7^. Other studies show that AI can be of benefit by decreasing inter-observer variations among physicians and increasing the sensitivity in finding lesions^8^.

Sibille et al. recently developed AI-based software for PET/CT images^9^. They included a mixture of lung cancer and lymphoma patients at different phases (29% during staging, 25% during treatment, and 46% after treatment), showing good performance in finding lesions. However, due to the few included newly diagnosed lymphoma patients in the test group, it is still unclear how their software performs in the detection of focal skeleton/BMU in this specific patient category.

Our aim was to develop an AI-based method for the detection of focal skeleton/BMU and quantification of diffuse BMU in patients with HL undergoing staging with FDG-PET/CT. The output of the AI-based method in a separate test set was compared to the image interpretation of ten physicians from different hospitals. Finally, the AI-based quantification of diffuse BMU was compared to manual quantification.

## Methods

### Patients

#### Training cohort

All 156 patients who had undergone a staging PET/CT between 2011 and 2016 at Sahlgrenska University Hospital with biopsy-proven HL were retrospectively included. All were newly diagnosed and un-treated patients. Two patients were excluded due to incomplete image sets, and one patient was excluded due to failed skeletal segmentation. The final group consisted of 153 patients (Table 1). These examinations were used in the development of the AI-based method.

**Table 1:** Study population.

	Training cohort	Test cohort
Number of patients	153	48
Female (%)	46	46
Median age (y), range	33, 11–85	35, 7–75

#### Test cohort

All 49 patients who had undergone staging with FDG-PET/CT between 2017 and 2018 at Sahlgrenska University Hospital with biopsy-proven HL were retrospectively included. All were newly diagnosed and un-treated patients. One patient was excluded due to a falsely reported injection time, and 48 patients were evaluated as the test cohort (Table 1). These examinations were used for the testing of the AI-based method and for inter-observer classifications.

#### Image acquisition

PET/CT data were obtained using an integrated PET/CT system (Siemens Biograph 64 Truepoint). The adult patients were injected with 4 MBq/kg 18F-FDG (maximum 400 MBq) and fasted for at least 6 h before the injection of FDG. The injected amount of radioactivity for children was according to the EANM Dosage Card (Version 5.7.2016). The standard accumulation time was 60 min. Images were acquired with 3 min per bed position from the base of the skull to the mid-thigh. PET images were reconstructed with a slice thickness of 5 mm and slice spacing of 3 mm with an iterative OSEM 3D algorithm (4 iterations and 8 subsets) and matrix size of 168 × 168. CT-based attenuation and scatter corrections were applied. A low-dose CT scan (64-slice helical, 120 kV, 30 mAs, 512 × 512 matrix) was obtained covering the same part of the patient as the PET scan. The CT was reconstructed using a filtered back-projection algorithm with a slice thickness and spacing matching those of the PET scan^10^.

### Artificial intelligence-based classification

A convolutional neural network (CNN) was used to segment the skeletal anatomy^11^. Based on this CNN, the bone marrow was defined by excluding the edges from each individual bone; more precisely, 7 mm was excluded from the humeri and femora, 5 mm was excluded from the vertebrae and hip bones, and 3 mm was excluded from the remaining bones.

#### Focal skeleton/bone marrow uptake

The basic idea behind our approach is that the distribution of non-focal BMU has a light tail and most pixels will have an uptake reasonably close to the average. There will still be variations between different bones. Most importantly, we found that certain bones were much more likely to have diffuse BMU than others. Hence, we cannot use the same threshold for focal uptake in all bones. At the other end, treating each bone individually is too susceptible to noise. As a compromise, we chose to divide the bones into two groups:

- “*spine*”—defined as the vertebrae, sacrum, and coccyx as well as regions in the hip bones within 50 mm from these locations, i.e., including the sacroiliac joints.
- “*other bones*”—defined as the humeri, scapulae, clavicles, ribs, sternum, femora, and the remaining parts of the hip bones.

For each group, the focal standardized uptake values (SUVs) were quantified using the following steps:

1. *Threshold computation.* A threshold (THR) was computed using the mean and standard deviation (SD) of the SUV inside the bone marrow. The threshold was set to
  $$THR = {\text{SUV}}_{{{\text{mean}}}} + 2{\text{SD}}.$$
2. *Abnormal bone region.* The abnormal bone region was defined in the following way:
  Only the pixels segmented as bone and where SUV > THR were considered. To reduce the issues of PET/CT misalignment and spill over, a watershed transform was used to assign each of these pixels to a local maximum in the PET image. If this maximum was outside the bone mask, the uptake was assumed to be leaking into the bone from other tissues and was removed. Finally, uptake regions smaller than 0.1 mL were removed.
3. *Abnormal bone SUV quantification*. The mean squared abnormal uptake (MSAU) was first calculated as
  $${\text{MSAU}} = {\text{mean}}\;{\text{of}}\;\left( {{\text{SUV}}{-}{\text{THR}}} \right)^{2} \;{\text{over}}\;{\text{the}}\;{\text{abnormal}}\;{\text{bone}}\;{\text{region}}{.}$$
  To quantify the abnormal uptake, we used the total squared abnormal uptake (TSAU), rather than the more common total lesion glycolysis (TLG). We believe TLG tends to overestimate the severity of larger regions with moderate uptake. TSAU will assign a much smaller value to such lesions, reflecting the uncertainty that is often associated with their classification. Instead, TSAU will give a larger weight to small lesions with very high uptake. This reflects both the higher certainty with respect to their classification and the severity typically associated to very high uptake.
  $${\text{TSAU}} = {\text{MSAU}} \times ({\text{volume}}\;{\text{of}}\;{\text{the}}\;{\text{abnormal}}\;{\text{bone}}\;{\text{region}}).$$

This calculation leads to two TSAU values; one for the “spine” and one for the “other bones”. As the TSAU value can be nonzero even for patients without focal uptake, cut-off values were tuned using the training cohort. The AI method was adjusted in the training group to have a positive predictive value of 65% and a negative predictive value of 98%. For the “spine”, a cut-off of 0.5 was used, and for the “other bones”, a cut-off of 3.0 was used. If one of the TSAU values was higher than the corresponding cut-off, the patient was considered to have focal uptake.

#### Diffuse bone marrow uptake

**Figure 1:**
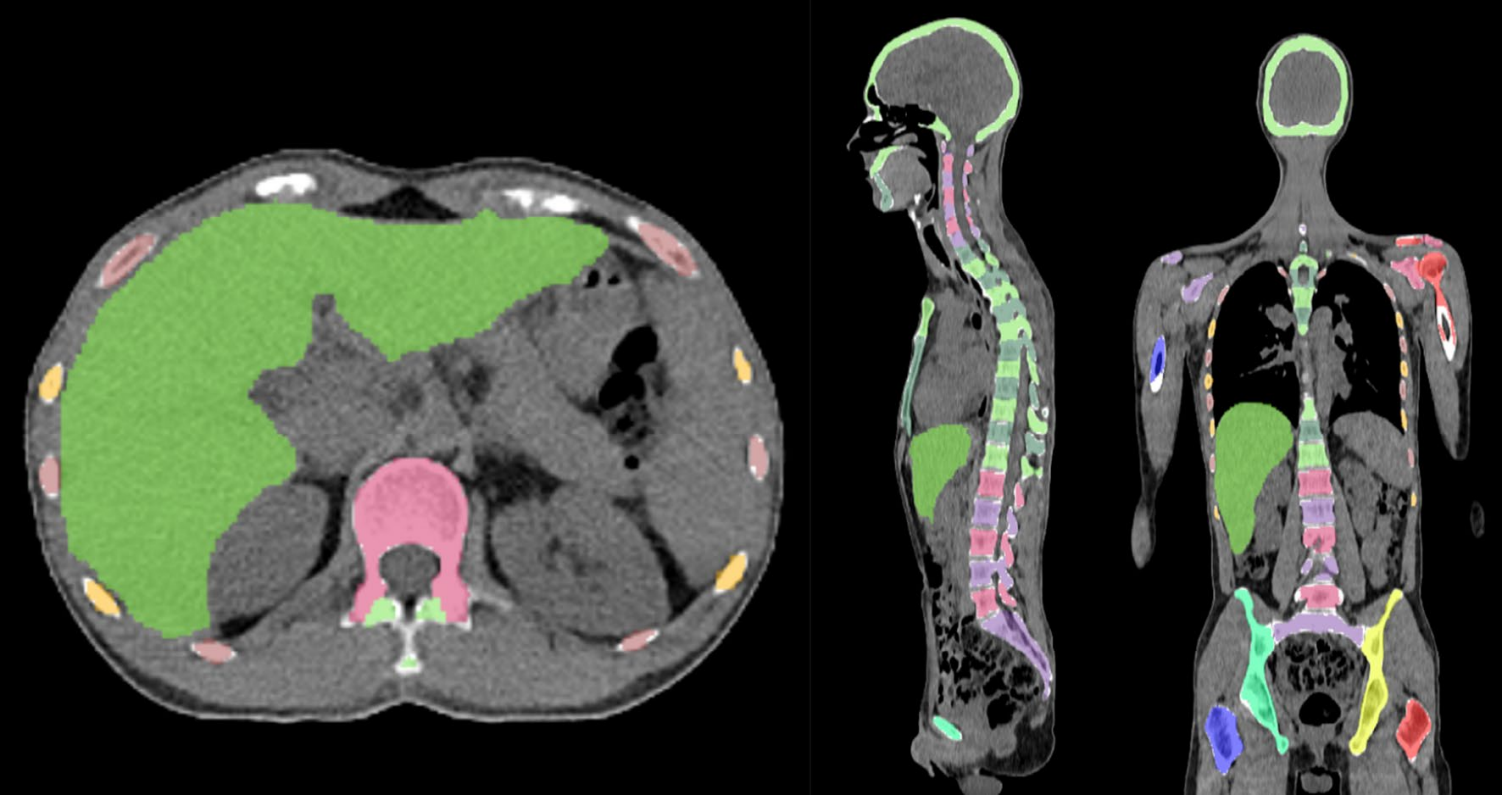
CT: Example of bone and liver segmentations performed by the artificial intelligence-based method.

The SUV_median_ in the vertebral bone marrow was automatically computed and compared to the median uptake in the liver. The latter was also segmented using a CNN according to^10^. Figure 1 shows an example. If the ratio between the median “spine” BMU and liver uptake was greater than 1.0, the patient was considered to have diffuse BMU^1^.

Quality control was performed in the test patients regarding the automated AI calculations of diffuse BMU. An experienced technologist manually placed the region of interest (ROI) in the marrow area of lumbar vertebrae L3/L4 and at the upper homogenous right part of the liver (excluding the edges). These regions were chosen according to the study by Pedersen et al.^1^.

### Image interpretations

#### Training

The original interpretation of the PET/CT examinations was performed by a nuclear medicine physician and a radiologist, who wrote the final report sent to the referring department. A trained technologist extracted information regarding skeleton and/or bone marrow involvement from these PET/CT reports and from the digital medical records. All the cases with focal or suspicious focal uptake in skeletal and/or bone marrow were reviewed again by a nuclear medicine specialist.

#### Test

Ten nuclear medicine physicians with 2–12 years of experience in PET/CT working in three different hospitals (two in Sweden (Malmö/Lund and Gothenburg) and one in India (Chandigarh)) were invited to participate. They separately classified the 48 FDG-PET/CT images regarding diffuse uptake in bone marrow and focal uptake in skeletal/bone marrow in the following four categories^1^.

- Low diffuse bone marrow uptake and no focal lesion(s)
- Low diffuse bone marrow uptake and focal lesion(s)
- High diffuse bone marrow uptake and no focal lesion(s)
- High diffuse bone marrow uptake and focal lesion(s)

The cases were presented in a different computer-generated randomized order to each physician. Information regarding sex, age, and investigations involving untreated staging HL patients was provided. The physicians were instructed to classify the cases as they normally do in the clinical setting. The review process was performed using RECOMIA software (recomia.org), and every case was presented with CT images, PET images, fused PET/CT images, and MIP images. The interpreter was also able to shift between sagittal, coronal and transverse planes. The PET images could be displayed in different colours with the images scaled to an upper SUV threshold of 5, and the latter could also be changed. The CT images could be shifted to the skeleton window.

The study was approved by the ethics committee at Gothenburg University, and the need for written informed consent was waived (#2019–01,274). We certify that the study was performed in accordance with the ethical standards laid down in the 1964 Declaration of Helsinki and its later amendments.

#### Statistical analyses

The percentage agreement (PA) and Kappa were used in the inter-observer comparisons between physicians for the classifications of both focal skeletal/BMU and diffuse BMU. Kappa takes into account chance agreement, and some suggested interpretation guidelines are as follows; values < 0 indicate no agreement, values between 0 and 0.20 indicate slight agreement, values between 0.21 and 0.40 indicate fair agreement, values between 0.41 and 0.60 indicate moderate agreement, values between 0.61 and 0.80 indicate substantial agreement, and values between 0.81 and 1 indicate almost perfect agreement^12^.

## Results

### Focal uptake

**Figure 2:**
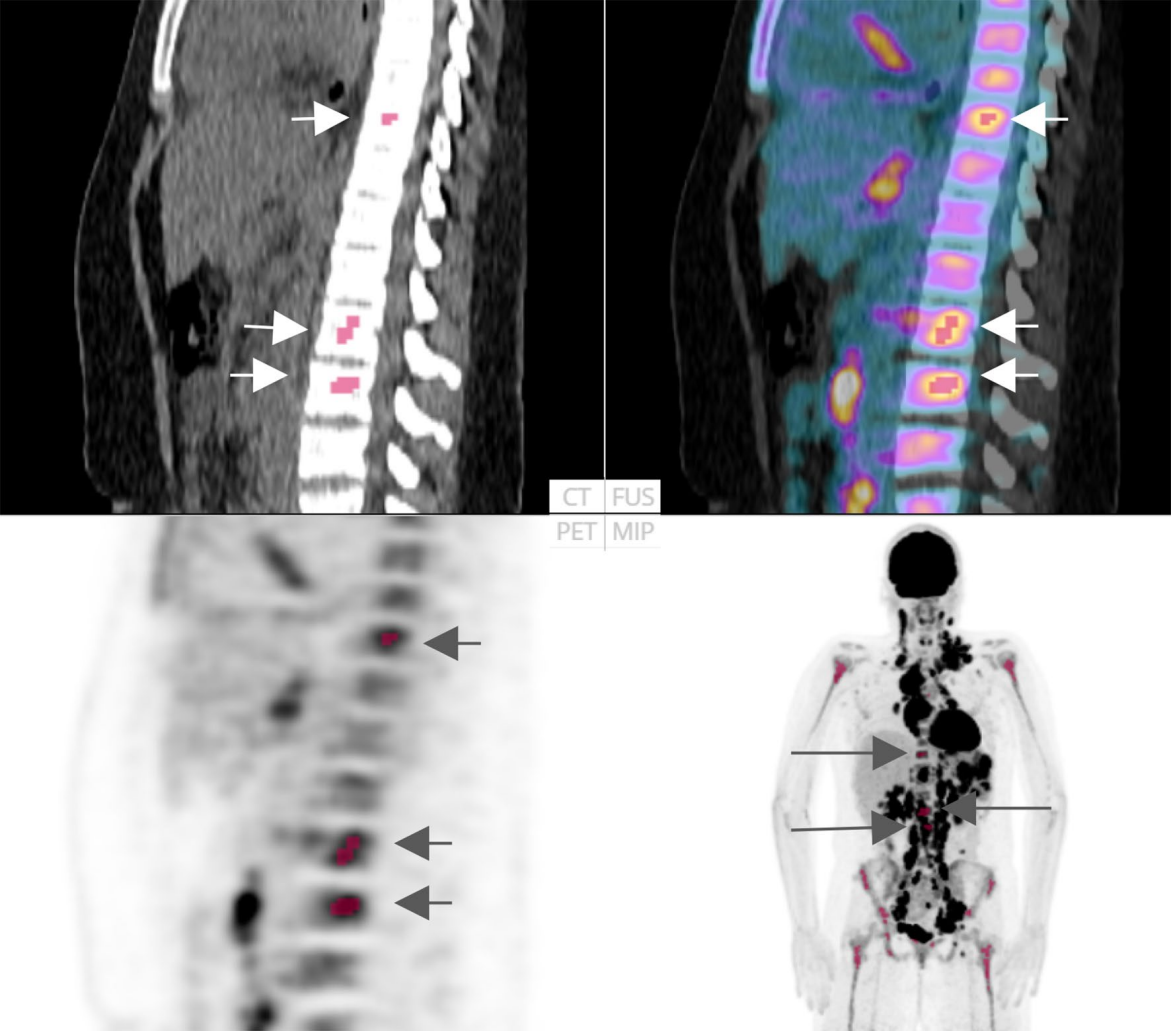
FDG-PET/CT: Example of a patient classified as having focal uptake in skeleton/bone marrow by the artificial intelligence-based method (highlighted in red and by arrows) and by the majority (8/10) of the physicians.

Fourteen of the 48 cases were classified as having focal skeleton/BMU by the AI-based method. The majority of physicians classified 7/48 cases as positive and 41/48 cases as negative for having focal skeleton/BMU. The majority of the physicians agreed with the AI method in 39 of the 48 cases. An example can be seen in Fig. 2. Six of the seven positive cases (86%) identified by the majority of physicians were identified as positive by the AI method, while the seventh was classified as negative by the AI method and by three of the ten physicians.

Thirty-three of the 41 negative cases (80%) identified by the majority of physicians were also classified as negative by the AI method. In seven of the remaining eight patients, 1–3 physicians (out of the ten total) classified the cases as having focal uptake, while in one of the eight cases none of the physicians classified it as having focal uptake. These findings indicate that the AI method has been developed towards high sensitivity, which is necessary to highlight suspicious uptake.

The pairwise agreement between the ten physicians resulted in 45 unique pairs. The mean PA was 85% (range 79–94%), and the mean Kappa was 0.51 (range 0.25–0.80) between the paired physicians, regarding the presence or absence of focal skeleton/BMU. Examples of different Kappa values can be found in Tables 2–4.

**Table 2:** Frequency table showing the pairs of observers with the lowest Kappa (0.25) regarding the interpretations of focal skeletal/bone marrow uptake. Grey fields indicate agreement between physicians A and B.

Focal		B	
		No	Yes
A	No	35	4
	Yes	6	3

**Table 3:** Frequency table showing the pairs of observers with the median Kappa (0.54) regarding the interpretation of focal skeletal/bone marrow uptake. Grey fields indicate agreement between physicians C and D.

Focal		D	
		No	Yes
C	No	35	2
	Yes	5	6

**Table 4:** Frequency table showing the pairs of observers with the highest Kappa (0.80) regarding the interpretation of focal skeletal/bone marrow uptake. Grey fields indicate agreement between physicians E and F.

Focal		F	
		No	Yes
E	No	37	0
	Yes	3	8

### Diffuse bone marrow uptake

**Figure 3:**
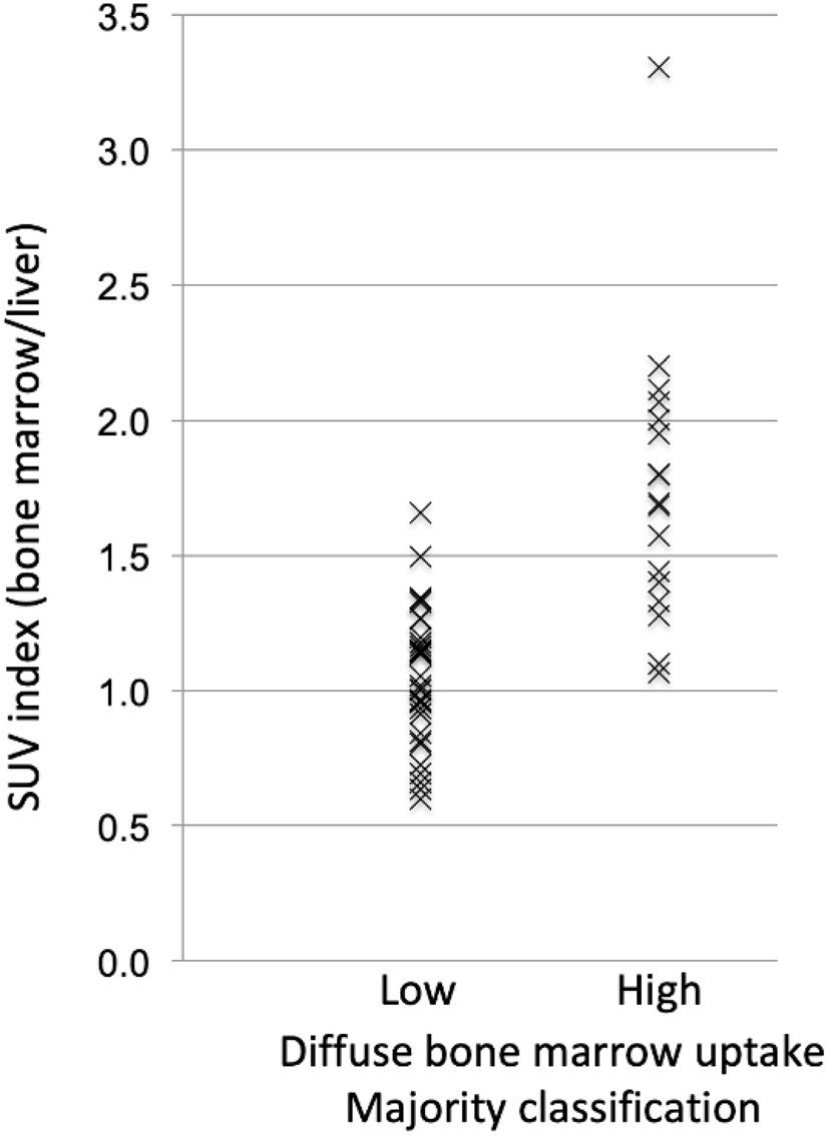
SUV index: (spine marrow uptake/liver) calculated by the artificial intelligence-based method compared with the physicians’ majority classifications of diffuse bone marrow uptake.

The automatic AI calculations of the SUV index, i.e., the median spine marrow uptake/liver, indicated that 32 patients had an index > 1.0 (high diffuse BMU), while 16 patients had an index of 1.0 or below (low diffuse BMU). In 33 (69%) cases, most of the physicians agreed with the AI method. In one case, half of the physicians agreed with the AI method, and in 14 cases, most of the physicians disagreed with the AI method regarding high versus low BMU. These 14 cases were classified as low diffuse BMU by most of the physicians (Fig. 3).

The agreement between the ten physicians (45 unique pairs) concerning high versus low diffuse BMU resulted in an average PA of 70% (range 21–88%), with a mean Kappa value of 0.41 (range 0.03–0.68).

**Figure 4:**
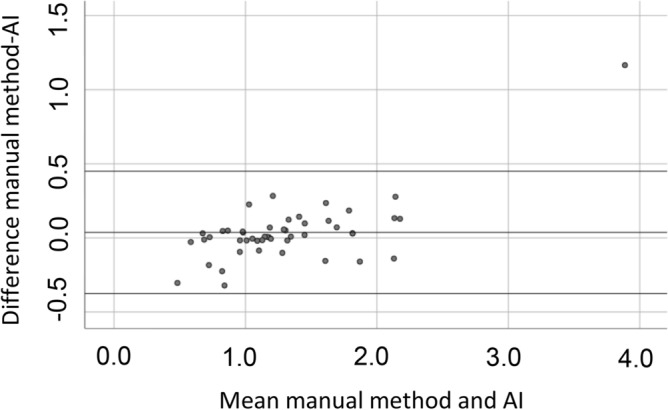
Bland–Altman plot: comparison between the SUV index calculated by the artificial intelligence (AI)-based method versus the manual method.

Figure 4 shows a Bland–Altman plot comparing the AI-automated SUV index calculations versus the manual method regarding high or low BMU. The graph indicates that the AI and the manual SUV index calculations are relatively comparable except for one outlier. This outlier had focal bone marrow involvement predominantly located in L4, why the SUV index with the manual method resulted in a much higher value compared with the AI method that takes into account the SUV_median_ value in the whole spine bone marrow. Table 5 shows the larger volumes used by the AI method compared with the manual ROI method in the SUV index calculations.

**Table 5:** Volumes used by the artificial intelligence (AI)-based method compared with the manual method for the SUV index calculations. ROI = Region of interest.

	AI (mL)	Manual ROI (cm^3^)
Spine bone marrow: median (range)	161 (32–298)	2 (1–3)
Liver: median (range)	1523 (607–2800)	14 (8–28)

If diffuse BMU is considered a dichotomous variable, i.e., high versus low, then both the AI versus manual methods were in concordance with each other in all cases except in one (SUV index 0.9 with the AI method versus 1.1 with the manual method).

## Discussion

The present study demonstrates that an AI-based method can be developed to highlight suspicious focal skeleton/BMU in HL patients staged with FDG-PET/CT. This AI-based method can also objectively provide results regarding high versus low BMU by calculating the SUV_median_ in the spine marrow and the liver. We have also demonstrated that inter-observer variations regarding both focal and diffuse BMU are moderate (mean Kappa 0.51 and 0.41, respectively) among the group of nuclear medicine physicians with 2–12 years of experience working at different hospitals. Finally, our results show that the automated method regarding diffuse BMU is comparable to the manual ROI method.

Pedersen et al. emphasize the importance of finding focal bone lesions (uni- or multifocal) since both progression-free survival rate and overall survival significantly indicate poorer prognosis compared with the non-bone lesion group^1^. Earlier studies report high inter-observer agreement (Kappa 0.84 and 0.74–0.86, respectively) among physicians, which differs from our results (mean Kappa 0.51)^13,14^. Zwarthoed et al. and Hofman et al. included two to three physicians interpreting 180 and 100 patients, respectively. No information was given in the former study regarding the experience levels or whether the observers were from the same institution, while in the latter study, all the physicians were experienced they seemingly worked at the same hospital. It is known that training and continuous discussions among colleagues improve inter-observer agreement^15^. The moderate agreement found in our study, including ten physicians from different centres interpreting 48 cases, is probably more generalizable since not all the physicians interpreting PET/CT were experienced or were working in the same institution.

The need to develop an AI-based method is not to replace physicians but rather to help them by taking advantage of the rapid calculations performed by AI. The results showed that the majority of the physicians agreed with the AI interpretations regarding focal uptake in 81% (39/48) of the cases but disagreed in nine of the cases. Eight of these nine cases were classified as positive by the AI method, and in seven of those 1–3 (of the 10) physicians interpreted the images as positive for focal uptake. We felt that it was important to shift the system towards high sensitivity to avoid missing suspicious findings. The strength of physicians, on the other hand, is that they can dismiss uptake due to benign conditions such as degeneration in the spine or uptake in brown fat adjacent to bones; i.e., they have a higher specificity than the AI-based method.

Sibillie et al. developed an automated method for the classification of lung cancer and lymphoma showing high performance with an area under the receiver operating characteristic curve of 0.98^9^. However, their included patients had, apart from different kinds of cancers, also a mix of different lymphoma types examined at different phases (staging, during treatment and after treatment) which makes a direct comparison with our study, which focused solely on staging HL patients, difficult. Another limitation in their study was that they used the same physicians for the segmentation of lesions both for training, validation, and testing. Therefore, it is likely that the classifications tend to agree more between training and testing, and an independent gold standard for the test group would be preferable.

**Figure 5:**
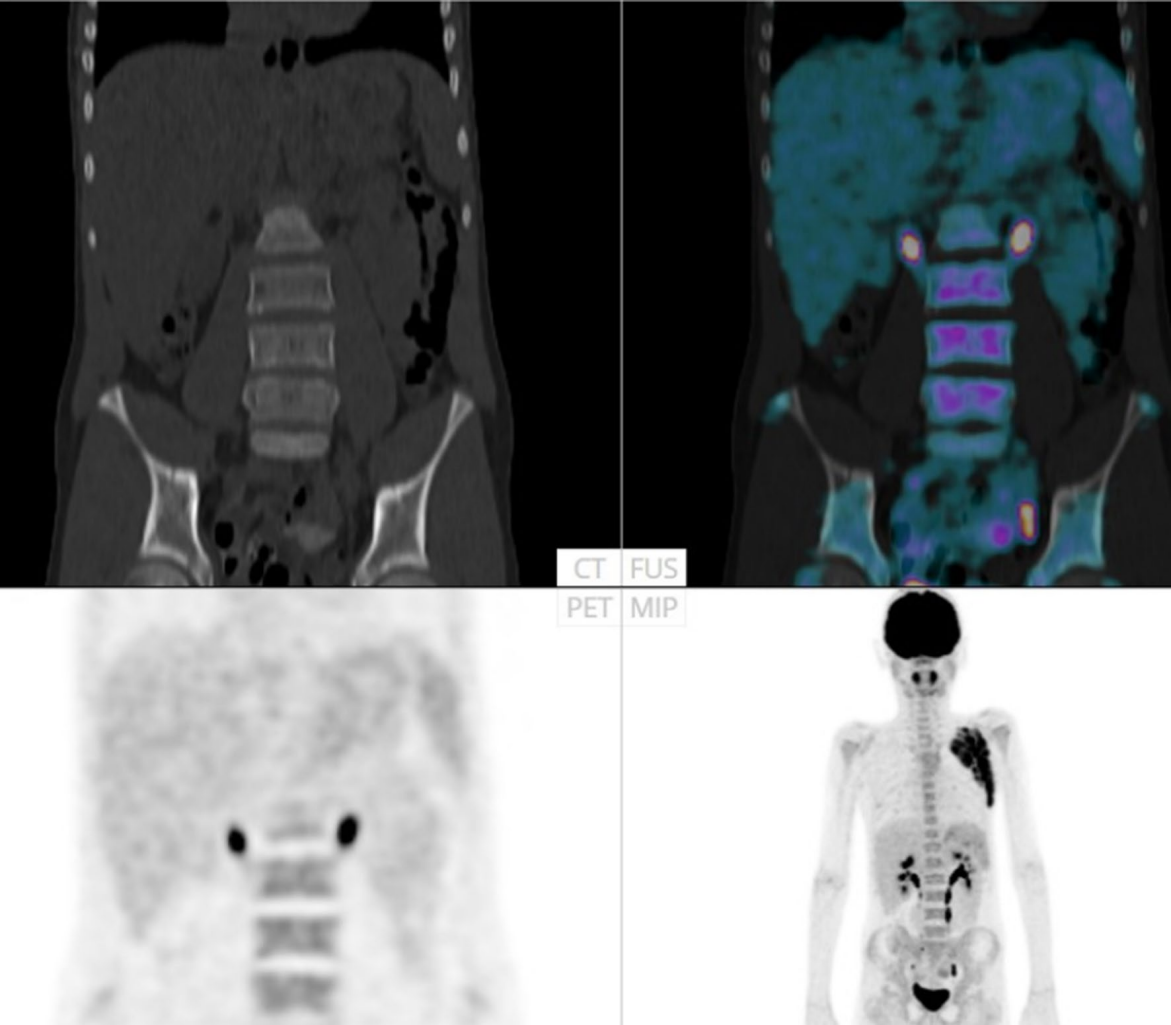
FDG-PET/CT: Example of a patient classified as having high diffuse bone marrow uptake (BMU) by the artificial intelligence-based method and by the manual method (SUV_index_ of 1,7 by both methods), while the majority (6/10) of the physicians classified this case as having low BMU.

Earlier studies revealed that diffusely increased BMU is likely to be reactive/inflammatory, i.e., correlate with lower haemoglobin levels and higher leukocyte counts, and should not be considered a risk factor^1,13^. It is, however, a common practice that physicians mention high diffuse BMU in the final report. Pedersen et al. found that physicians tend to underestimate high BMU when the visual method is used, i.e., comparing the bone marrow with the liver^1^. This finding is in concordance with our results, that the most common cause of disagreement between the physicians and AI (29%, 14/48 cases) was due to the underestimation of the BMU. Figure 5 shows an example. Pedersen et al. recommend that if physicians find it necessary to comment on this task, then the comment should be based on objective calculations of SUV in, for example, L3/L4 and the upper part of the liver. If our automated AI calculations were treated as a continuous variable, then the results were relatively comparable with the manual ROI method (Figs. 3 and 4), and if treated as a dichotomous variable (high versus low BMU), the two methods agreed in all cases except in one.

Before applying this novel AI-based method in the clinical setting, it should be further evaluated in a new group of HL patients from other hospitals and examined with other PET/CT devices. The training of the AI method is also crucial. We used the final report of the PET/CT examination sent to the refereeing department and extracted information regarding bone and bone marrow involvement from the medical record. However, we did not define the ground truth for the test group. Instead, the physicians’ interpretations were compared with each other resulting in moderate agreement both for focal uptake and with even lower Kappa for diffuse BMU. This finding indicates that interpretations of this kind are difficult. Collaborations and further studies with other centres providing similar patient material are welcomed.

Achieving equal healthcare is essential; i.e., interpretations of the same diagnostic images should be similar independent of the experience of the reporting physicians. Our aim was to develop an AI-based method to highlight suspicious focal uptake in skeleton/bone marrow and provide a result for high/low diffused BMU. The future goal is to systemize interpretations and minimize inter-observer variations. The AI-based method is independent of temporary human tiredness, disturbance, and type of department such as a university hospital setting with many colleagues available for discussions or a regional hospital. The next step is to invite the same physicians to interpret the same PET/CT images, this time also considering the AI-based suggestions regarding focal and diffuse uptake in skeleton/bone marrow.

Even though high BMU should not be considered a risk factor^1,13^, the lowest Kappa among paired observers was 0.03, i.e., agreement equivalent to chance. Our AI-based method can objectively provide a value that corresponds relatively well with the manual ROI method (Fig. 4). An explanation of the difference between the two methods could be that the AI method uses the bone marrow volume for the whole spine and the liver, excluding the edges, while the manual ROI method is restricted to three slices (two in L3/L4 and one in the liver). Finally, we used SUV_median_ instead of SUV_max_ to minimize extreme values due to focal lesion uptake.

## Conclusions

The present study demonstrates that an AI-based method can be developed to highlight suspicious focal skeleton/BMU in HL patients staged with FDG-PET/CT. This AI-based method can also objectively provide results regarding high versus low BMU by calculating the SUV_median_ value in the whole spine marrow and the liver. Additionally, the study also demonstrated that inter-observer agreement regarding both focal and diffuse BMU is moderate among nuclear medicine physicians with varying levels of experience working at different hospitals. Finally, our results show that the automated method regarding diffuse BMU is comparable to the manual ROI method.

## Data Availability

The datasets generated during and/or analysed during the current study are available from the corresponding author on reasonable request.

## References

[1] Pedersen MA (2019). Focal skeletal FDG uptake indicates poor prognosis in cHL regardless of extent and first-line chemotherapy. Br. J. Haematol..

[2] El-Galaly TC (2014). Impact of 18F-fluorodeoxyglucose positron emission tomography/computed tomography staging in newly diagnosed classical Hodgkin lymphoma: fewer cases with stage I disease and more with skeletal involvement. Leuk. Lymphoma..

[3] Barrington SF (2016). PET-CT for staging and early response: results from the Response-Adapted Therapy in Advanced Hodgkin Lymphoma study. Blood.

[4] Cheson BD (2014). Recommendations for initial evaluation, staging, and response assessment of Hodgkin and non-Hodgkin lymphoma: the Lugano classification. J. Clin. Oncol..

[5] El-Galaly TC (2012). Routine bone marrow biopsy has little or no therapeutic consequence for positron emission tomography/computed tomography-staged treatment-naive patients with Hodgkin lymphoma. J. Clin. Oncol..

[6] Hosny A, Parmar C, Quackenbush J, Schwartz LH, Aerts HJWL (2018). Artificial intelligence in radiology. Nat. Rev. Cancer..

[7] Reza M (2019). Automated bone scan index as an imaging biomarker to predict overall survival in the Zometa European Study/SPCG11. Eur. Urol. Oncol..

[8] Sadik M, Suurkula M, Höglund P, Järund A, Edenbrandt L (2009). Improved classifications of planar whole-body bone scans using a computer-assisted diagnosis system: A multicenter, multiple-reader, multiple-case study. J. Nucl. Med..

[9] Sibille L (2020). ^18^F-FDG PET/CT uptake classification in lymphoma and lung cancer by using deep convolutional neural networks. Radiology.

[10] Sadik M (2019). Automated quantification of reference levels in liver and mediastinal blood pool for the Deauville therapy response classification using FDG-PET/CT in Hodgkin and non-Hodgkin lymphomas. Clin. Physiol. Funct. Imaging.

[11] Trägårdh E (2020). RECOMIA-a cloud-based platform for artificial intelligence research in nuclear medicine and radiology. EJNMMI. Phys..

[12] Landis JR, Koch GG (1977). The measurement of observer agreement for categorical data. Biometrics.

[13] Zwarthoed C (2017). Prognostic value of bone marrow tracer uptake pattern in baseline PET scans in hodgkin lymphoma: Results from an international collaborative study. J. Nucl. Med..

[14] Hofman MS, Smeeton NC, Rankin SC, Nunan T, O'Doherty MJ (2009). Observer variation in interpreting 18F-FDG PET/CT findings for lymphoma staging. J. Nucl. Med..

[15] Ceriani L (2017). Improves the interobserver agreement of the expert positron emission tomography review panel in primary mediastinal B-cell lymphoma: Interim analysis in the ongoing international extranodal lymphoma study group-37 study. Hematol. Oncol..

